# Overexpression of High Mobility Group A1 Protein in Human Uveal Melanomas: Implication for Prognosis

**DOI:** 10.1371/journal.pone.0068724

**Published:** 2013-07-23

**Authors:** Yi Qu, Yupeng Wang, Jinlan Ma, Yue Zhang, Nana Meng, Hao Li, Yan Wang, Wenbin Wei

**Affiliations:** 1 Department of Ophthalmology, Qilu Hospital of Shandong University, Jinan, China; 2 Shandong University School of Medicine, Jinan, China; 3 Department of Pathology, Qilu Hospital of Shandong University, Jinan, China; 4 Beijing Tongren Hospital, Capital Medical University, Beijing Tongren Eye Center, Beijing Ophthalmology and Visual Science Key Laboratory, Beijing, China; Weizmann Institute of Science, Israel

## Abstract

There is increasing evidence that the high mobility group A1 (HMGA1) protein, which functions as a transcriptional master regulator, plays critical roles in tumor progression. We evaluated HMGA1 expression in 89 primary uveal melanomas (UM) by immunohistochemistry to determine the clinicopathological and prognostic value of HMGA1 in UM after adjusting for other prognostic variables. Nuclear expression of HMGA1 was detected in 44% UMs. High expression levels of HMGA1 were more frequent in UMs with high levels of epithelioid cell pattern, mitoses count, and Ki67 labeling index (*P* = 0.025, *P*<0.0001, *P* = 0.0018; respectively), and HMGA1 expression levels were directly correlated with Ki67 labeling indexes and mitoses counts (R = 0.31, *P* <0.0001; R = 0.27, *P*<0.0068; respectively). High expression of HMGA1 was also independently associated with an increased risk of distant metastases as determined using the Cox proportional hazards regression model (multivariate hazard ratio: 3.44; 95% confidence interval: 1.56–7.60; log rank *P* = 0.0022). Moreover, high HMGA1 expression was associated with shorter UM-specific survival (multivariate hazard ratio: 2.41; 95% confidence interval: 1.10–5.53; log rank P = 0.041). These findings suggest that high levels of HMGA1 are associated with adverse clinical outcomes in UM patients and that further evaluation of HMGA1 as a potential therapeutic target in UM is warranted.

## Introduction

Uveal melanomas (UMs), which arise from neural crest-derived melanocytes within the choroidal plexus of the eye including iris, ciliary body, and choroid are the most common primary intraocular malignant tumors in adults and the most common noncutaneous melanomas. Although they comprise approximately 5% of all diagnosed melanoma cases, their incidence is only 6 per 1 million per year among white and even less among non-white races [1]–[3]. Nevertheless, UMs account for 13% of all deaths from melanoma [4]. In fact, despite the increased diagnostic accuracy and the development of conservative and effective early local treatments of primary intraocular tumors including plaque radiotherapy and photon beam therapy, up to 50% of UM patients die within 15 years of diagnosis from metastases, which almost exclusively involve the liver [3], [5], [6]. Once metastases are diagnosed, the patients' survival time averages no more than 5 to 8 months [7], [8]. presumably due to silent hematogenous systemic micrometastases that occur prior to the diagnosis of clinically evident ocular disease [9], [10]. As no effective therapies are currently available to decrease the risk of metastases, efforts have been made to identify high-risk patients in order to target systemic adjuvant therapy aimed at preventing or delaying metastatic disease.

Many clinicopathological factors including largest basal tumor diameter, age, the presence of epithelioid melanoma cells, closed vascular loops, mitotic activity, nodular growth and extracellular matrix patterns [11]–[15] have been found to be associated with increased risk of developing metastatic disease in UM. Regarding genetic evaluation, chromosome 3 monosomy, mutations of *GNAQ* or *GNA11* and breast cancer 1-associated protein (*BAP1*) have all been found to impart poor prognosis [16]–[19]. However, none of the above parameters or genes can be considered as therapeutic targets in UM.

High mobility group (HMG) proteins interact with other proteins to alter chromatin structure and so affect gene expression. They are involved in diverse cellular processes, including cell cycle progression, embryologic development, neoplastic transformation, differentiation, apoptosis, cellular metabolism, and DNA repair. In most differentiated tissues of adults, however, they are absent or accumulate to only very low levels [20], [21]. Nevertheless, increasing evidence suggests that a member of this family of proteins, HMGA1, plays a critical role in tumor progression in diverse malignancies. Originally, HMGA1 was described to function as a potent oncogene in cultured cells [22]–[24] and transgenic mice [25], [26]. The expression level of HMGA1 protein was found to be linked to the highly malignant phenotype of human cancers and a poor prognostic indicator, for instance for retinoblastomas [27]–[29]. It has been suggested that HMGA1 might serve not only as a potential biomarker but also as a therapeutic target for advanced malignancies [27]–[29] and metastatic progression [30], [31]. Hence, we speculated that HMGA1 may also play a key role in UM prognosis and as potential target for therapeutic intervention in UM. MIB-1 LI recognizes the nuclear antigen (Ki-67), which makes it an excellent marker of cells in the proliferative phase. In addition to being widely used to assess tumor proliferation rate, MIB-1 LI has prognostic value [32]. In our previous study [28], high MIB-1 LI expression levels were associated with poor prognosis in retinoblastomas, and the increased expression of HMGA1 and HMGA2 correlated with the MIB-1 LI.

In the present study, we explored the prognostic value of HMGA1 by an immunohistochemical analysis in a series of UM cases. The data were then correlated with the relevant clinical information and with the labeling index for the cell proliferation marker Ki67/MIB-1. Furthermore, we discuss the potential of HMGA1 protein as a prognostic marker of UM progression and possible approaches for inhibition of HMGA activity in UM.

## Materials and Methods

### Ethics statement

This study conformed to the Declaration of Helsinki and was approved by the ethics committee of Qilu Hospital of Shandong Unversity or Beijing Tongren Hospital. All patients had previously given their written informed consent for experimental research on residual tumor tissues available after histopathologic and cytogenetic analyses. The consent procedure was approved by the ethics committee of Qilu Hospital of Shandong Unversity or Beijing Tongren Hospital.

### Patients and tumor samples

Eighty-nine primary UM lesions were obtained after enucleation with complete clinical data between 1998 and 2006. Full ophthalmologic examinations were conducted. Systemic clinical examinations were performed routinely with preoperative and postoperative liver function tests, chest X-ray and abdominal ultrasonography. Computed tomography or magnetic resonance imaging was used to confirm metastases that were suspected in screening examinations. All patients were evaluated at the ocular oncology clinic of Qilu hospital or Beijing Tongren hospital. The enucleation was the standard treatment for UM in China during the indicated period.

The duration of the follow-up period was calculated from the date of UM diagnosis to the date of death or last follow-up. For the purpose of the current study, patients were followed up until death or January 1^st^ 2012, whichever came first. Eighty-nine patients with complete follow-up data have been examined in survival analyses. This study conformed to the Declaration of Helsinki and was approved by the ethics committee of the appropriate institutes. All patients had previously given their informed consent for experimental research on residual tumor tissues available after histopathologic and cytogenetic analyses.

### Histopathologic examination

Histological details were obtained from the pathology reports and were reviewed and confirmed by one pathologist unaware of other data. The diagnosis of melanoma was confirmed using sections stained for hematoxylin and eosin (H&E) and/or Melan A [33]. The tumors were histologically examined for cell type, localization, size, mitosis frequency, necrosis, and scleral invasion. Spindle and epithelioid cell types were assessed using the modified Callender system [34]. Extravascular matrix patterns were assessed using the periodic acid-Schiff reagent without hematoxylin counterstaining, and the sections were viewed under a green filter [35]. The mitotic count was measured by counting the number of mitoses in 40 high-power fields (HPF) in the H&E sections [33].

### Immunohistochemistry

Histological sections of formalin-fixed paraffin-embedded samples were analyzed for the presence of Ki67 and HMGA1 by the labeled streptavidin-biotin method. After deparaffinization and antigen retrieval using an autoclave oven technique, sections were incubated at 4°C overnight and incubated with rabbit polyclonal anti-HMGA1 antibody (1∶50; ab4078, Abcam Inc, Cambridge, MA) and Ki67 antigen mouse monoclonal antibody (1∶75, DakoCytomatin, Glostrup, Denmark) at 4°C. Antigen-antibody complexes were detected by the cobalt-3, 3′-diaminobenzidine reaction. Squamous cell carcinomas known to be positive for HMGA1 expression were used as positive controls [36]. Sections incubated in phosphate-buffered saline without the primary antibody served as negative controls.

Staining for HMGA1and Ki67 was considered positive when it was nuclear. Images of several high-power fields (HPF; ×400) were captured from regions with different staining intensities, including high, moderate, low, and negative staining for each case. The photographs were printed on plain paper, and a grid was drawn over them. A total of 1000 cells were counted and expressed as a percentage of tumor cells with positive nuclei. The percentage of HMGA1 positive tumor cells was scored on a scale from 0 to 4 (0, no staining; 1+, ≤10%; 2+, ≤30%; 3+, ≤50%; 4+, >50%). Then, the expression levels of HMGA1 were divided into two groups according to score: low (score: 0, 1+); high (score: 2+, 3+, and 4+) [36], [37]. The Ki67 labeling index (LI) was determined by counting the number of positive cells in a total of 800–1000 tumor cells observed in regions of highest staining (hot spot) at several HPF (×400). The results were expressed as a percentage of tumor cells with positive nuclei.

### Statistical analysis

Biostatistical analyses were performed with StatView 5.0.1 software (Abacus Concepts, Berkeley, CA). The Pearson χ^2^ test or Fisher's exact test was used to compare qualitative variables. P values were calculated by ANOVA for age. In order to determine the significance of the associations and differences among the different variables, the data were analyzed using the Mann-Whitney U test and χ2 test. The association among the expression levels of HMGA1 and Ki67 LI were analyzed by Pearson's correlation coefficient. The Kaplan-Meier method and log-rank test were used for survival analyses. Cox proportional hazards regression model was used to compute mortality hazard ratios and 95% confidence intervals (CIs). To control for confounding variables, we used multivariate Cox proportional hazards regression models. To assess independent association between HMGA1 expression and key severity markers (epithelioid cells, mitosis count and Ki67 LI), multivariate logistic regression analysis was done and odds ratio (OR) was adjusted for age and gender. Probability values (P) <0.05 were considered to be statistically significant.

## Results

### Clinical and histopathologic characterisation

The 89 patients, of which 49 (55%) were male and 40 female, had a mean age of 46±14.5 years. Their tumors had a mean largest basal diameter of 14±3.4 mm (range: 7.0–21.1 mm) and a mean height of 10.1±3.3 mm (range: 3.0–20 mm). Nodular growth pattern was observed in 42 (47%) cases. There were 17 (19%) cases with ciliary body involvement, 9 (10%) cases with optic disc involvement, and 29 (32%) cases with closed vascular loops. Extraocular spread was present in 11 (12%) cases and epithelioid cell pattern was detected in 13 (15%) cases. High mitoses rates were found in 20 (24%) cases and high Ki67 labeling indexes (LIs) were detected in 19 (21%) cases (Table 1).

**Table 1:** Clinical, pathologic characteristics according to HMGA1 alterations in uveal melanoma.

		HMGA1 nuclear expression		
Clinical, pathologic features	Total N	High	Low	p value
	89	25 (28%)	64 (72%)	
Gender				0.55
Male, n (%)	49 (55%)	15 (60%)	34 (51%)	
Female, n (%)	40 (45%)	10 (40%)	30 (49%)	
Mean age at diagnosis ± SD	46.0±14.5	48.3±15.3	45.1±14.2	0.38
Laterality				0.52
Left eye, n (%)	38 (43%)	12 (48%)	26 (41%)	
Right eye, n (%)	51 (57%)	13 (52%)	38 (59%)	
Largest basal tumor diameter (mm)				
Mean (range)	14.0 (7–21)	14 (7–21)	13.9 (9–18)	0.98
<15, n (%)	59 (66%)	17 (68%)	42 (66%)	0.83
>15, n (%)	30 (34%)	8 (32%)	22 (34%)	
Tumor thickness (mm)				
Mean (range)	10.1 (3–20)	10.2 (3–20)	9.8 (6–15)	0.91
<10, n (%)	46 (52%)	14 (56%)	32 (50%)	0.61
>10, n (%)	43 (48%)	11 (34%)	32 (50%)	
Tumor growth pattern (Nodular)				0.39
Yes, n (%)	42 (47%)	10 (40%)	32 (50%)	
No, n (%)	47 (53%)	15 (60%)	32 (50%)	
Ciliary body involvement				0.64
Yes, n (%)	17 (19%)	4 (16%)	13 (20%)	
No, n (%)	72 (81%)	21 (84%)	51 (80%)	
Optic disc involvement				0.71
Yes, n (%)	9 (10%)	3 (13%)	6 (9%)	
No, n (%)	80 (90%)	22 (87%)	58 (91%)	
Closed loop				0.94
Yes, n (%)	29 (33%)	8 (32%)	21 (33%)	
No, n (%)	60 (67%)	17 (68%)	43 (67%)	
Extraocular spread				0.94
Yes, n (%)	11 (12%)	3 (13%)	8 (13%)	
No, n (%)	78 (88%)	22 (87%)	56 (87%)	
Epithelioid cells				0.025
Yes, n (%)	13 (15%)	7 (28%)	6 (9%)	
No, n (%)	76 (85%)	18 (72%)	58 (91%)	
Mitoses count / 40 HPF				<0.0001
≤4, n (%)	69 (76%)	11 (44%)	58 (91%)	
>4, n (%)	20 (24%)	14 (56%)	6 (9%)	
Ki67 labeling index				0.0018
≤2, n (%)	70 (79%)	14 (56%)	56 (87%)	
>2, n (%)	19 (21%)	11 (44%)	8 (13%)	

The mean follow-up time in the 89 patients that were analyzed for survival was 53±22 months (median  = 55 months; range: 8–120 months), and there were 25 distant metastases and 25 deaths from metastases. The median time from enucleation to the first clinical detection of distant metastases (liver and brain) was 30 months (range: 4–56 months). The median time from metastasis to death was 5 months (range: 2–9 months). In addition, there were 5 censors with follow-up data before the reference time horizon, January 2012. However, their follow-up time was longer than 8 years.

### HMGA1 expression and correlation with other characteristics in UM

**Figure 1:**
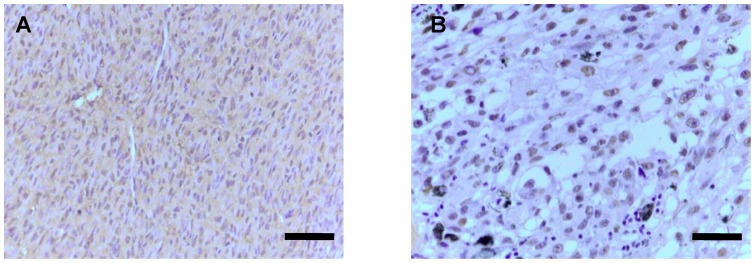
Expression of HMGA1 in UM. Detection of high mobility group A 1 (HMGA1) immunoreactivity in UM. **A**, HMGA1 expression in melanoma spindle cells (Scale bar – 100µm). **B**, HMGA1 expression in melanoma epithelioid cells (Scale bar – 50µm).

In uveal melanoma specimens, immunopositivity for HMGA1 showed a clear nuclear staining. Weak cytoplasmic localization was rarely observed. Nuclear staining was typically very intense, encompassing the entire nucleus of tumor cells, regardless of their specific type (Figure 1A, B). Nuclear expression of HMGA1 was detected in 39 uveal melanomas (44%), whereby 25 of them (28% of the total of 89 patient samples) expressed it a high levels (marked 4+, 3+ and 2+ as described in detail in Materials & Methods) (Table 1).

**Figure 2:**
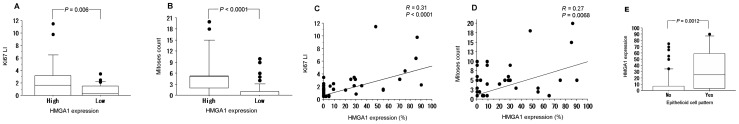
Correlation of HMGA1 expression levels and other characteristics in UM. Immunoreactivity levels of HMGA1, Ki67 LI and mitoses counts. In A, B, E, values are expressed as the median (horizontal line in each box), with the 25th and 75th percentiles (interquartile range, top and bottom of each box) and the 10th and 90th percentiles range (top and bottom of each bar). Dots indicate outliers. **A,** Ki67 LI was significantly higher in cases with high HMGA1 expression levels compared to cases with low expression. **B,** Mitosis counts were also significantly higher in HMGA1 high cases compared to HMGA1 low cases. Significant correlations have been detected between the expression of HMGA1 and Ki67 LI (**C**), and mitoses counts (**D**). **E,** HMGA1 expression levels were significantly higher in cases showing epithelioid cell pattern than in cases lacking this cell pattern.

High expression levels of HMGA1 were more frequent in UM with high levels of mitoses counts and high levels of Ki67 LI (*P*<0.0001, *P* = 0.0018, respectively; Table 1). Furthermore, the Ki67 LI and mitoses counts were significantly higher in UM with high levels of HMGA1 (mean 2.4% and 5.3%, respectively) than in those with low levels of HMGA1 (median 0.7% and 1%, respectively) (high versus low, *P* = 0.006; and *P*<0.0001, respectively) (Figure 2A, B; Mann-Whitney U test). Finally, the above correlations were confirmed using Pearson's correlation coefficient analysis (R = 0.31, *P*<0.0001; R = 0.27, *P*<0.0068, respectively) (Figure 2C, D; Pearson's correlation coefficient). In addition, in a multivariate logistic regression analysis, high level of mitoses counts was independently associated with high level of HMGA1 (Table S1). The frequency of high levels HMGA1 expression was higher in UM with epithelioid cell patterns (*P* = 0.025; Table 1) and the mean level of HMGA1 expression (%) was also significantly higher in these cases (*P* = 0.0012; Figure 2E; Mann-Whitney U test). Nevertheless, there were no significant correlations between HMGA1 expression levels and other clinicopathological characteristics (Table 1).

### Impact of HMGA1 on UM patient survival and association with distant metastases

**Figure 3:**
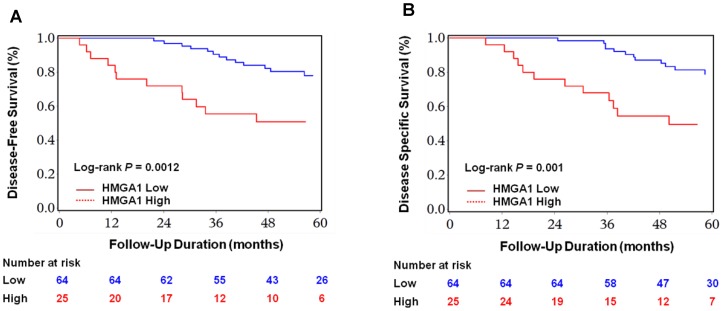
Kaplan-Meier curves for survival in UM patients. Survival of UM patients were estimated according to HMGA1 expression levels and Ki67 LI. Significant differences in disease-free survival rates were observed (**A, B**). Significant differences in disease-specific survival rates were also observed (**C, D**). P values were calculated with a log-rank test.

During the clinically disease-free survival period, high expression of HMGA1 was significantly correlated with increased risk of distant metastases using the log-rank test analysis (log rank P = 0.0012; Figure 3A). By univariate Cox regression, presence of epithelioid cell pattern (hazard ratio (HR), 3.02; 95% CI, 1.26–7.29; *P* = 0.013), high level of mitoses count (HR, 5.02; 95% CI, 2.31–9.31; *P* = 0.0012), high Ki67 LI (HR, 3.35; 95% CI, 1.51–6.48; *P* = 0.0034), and high level of HMGA1 expression (HR, 3.41; 95% CI, 1.55–7.50; *P* = 0.0023), were significantly associated with an increased risk of distant metastases (Table 2). By multivariate Cox regression (Table 2), both high mitoses count and high level of HMGA1 expression were significant prognostic predictors of distant metastases (multivariate HR, 4.17; 95% CI, 1.86–8.28; *P* = 0.005; multivariate HR, 3.44; 95% CI, 1.56–7.60; *P* = 0.0022; respectively).

**Table 2:** Clinicopathological features, tumor markers, HMGA1, and uveal melanoma patients' survival.

		Total N (%)	No. of events	Univariate HR (95% CI)	*P*	Multivariate HR^1^ (95% CI)	*P*
Disease-free survival							
Epithelioid cells	No	76 (85%)	18	1 (reference)		1 (reference)	
	Yes	13 (15%)	7	3.02 (1.26 to 7.29)	0.013	1.67 (0.68 to 4.25)	0.28
Mitoses count	≤4	69 (76%)	13	1 (reference)		1 (reference)	
	>4	20 (24%)	12	5.02 (2.31 to 9.31)	0.0012	4.17 (1.86 to 8.28)	0.005
Ki67 LI	≤2	70 (79%)	15	1 (reference)		1 (reference)	
	>2	19 (21%)	10	3.35 (1.51 to 6.48)	0.0034	2.12 (0.82 to 4.86)	0.13
HMGA1	Low	64 (72%)	13	1 (reference)		1 (reference)	
	High	25 (28%)	12	3.41 (1.55 to 7.50)	0.0023	3.44 (1.56 to 7.60)	0.0022
Disease specific survival							
Epithelioid cells	No	76 (85%)	18	1 (reference)		1 (reference)	
	Yes	13 (15%)	7	3.26 (1.27 to 7.32)	0.014	1.59 (0.61 to 4.12)	0.34
Mitoses count	≤4	18 (24%)	13	1 (reference)		1 (reference)	
	>4	57 (76%)	12	5.12 (3.39 to 10.02)	0.0014	4.15 (2.91 to 8.37)	0.006
Ki67 LI	≤2	70 (79%)	15	1 (reference)		1 (reference)	
	>2	19 (21%)	10	3.52 (1.56 to 7.81)	0.0025	1.99 (0.81 to 4.95)	0.15
HMGA1	Low	64 (72%)	13	1 (reference)		1 (reference)	
	High	25 (28%)	12	3.46 (1.67 to 8.16)	0.0013	2.41 (1.10 to 5.53)	0.041

Abbreviations: HR, hazard ratio; CI, confidence interval.

1 The multivariate Cox regression model initially included the HMGA1 expression variable (high or low), age of diagnosis, sex, largest basal tumor diameter, tumor thickness and epithelioid cell pattern.

### Association with melanoma-specific survival

For disease-specific survival, high level of HMGA1 was significantly associated with an increased risk of disease specific mortality using the log-rank test analyses (*P* = 0.001; Figure 3B). By univariate Cox regression, presence of epithelioid cell pattern (HR, 3.26; 95% CI, 1.27–7.32; *P* = 0.014), high level of mitoses count (HR, 5.12; 95% CI, 3.39–10.02; *P* = 0.0014), high Ki67 LI (HR, 3.52; 95% CI, 1.56–7.81; *P* = 0.0025), and high level of HMGA1 expression (HR, 3.46; 95% CI, 1.67–8.16; *P* = 0.0013), were significantly associated with an increased risk of disease-specific mortality (Table 2). By multivariate Cox regression (Table 2), both high mitoses count and high level of HMGA1 expression were significant prognostic predictors of worse outcome (multivariate HR, 4.15; 95% CI, 2.91–8.37; *P* = 0.006; multivariate HR, 2.41; 95% CI, 1.10–5.53; *P* = 0.041; respectively).

## Discussion

To our knowledge, this is the first study reporting HMGA1 expression in UM. In fact, nuclear expression of HMGA1 was frequent in UM, and high expression levels of HMGA1 were more prevalent in UM with high levels of epithelioid cell pattern, mitoses counts, and Ki67 LI. The latter is a cell-proliferation marker that is considered an independent prognostic parameter for UM [38]. As originally speculated high expression levels of HMGA1 protein were associated with highly malignant phenotypes of UM and hence can be considered as a marker for poor prognosis.

As metastatic UM is typically resistant to therapy [39], [40], it is critical to find methods that allow early detection of primary melanoma and that may serve to reliably predict the course of the disease. Genomic changes [16]–[19], including monosomy 3, *BAP1* and somatic mutations in *GNA11*, are associated with metastic UM. Regarding harvesting tissue for genetic evaluation, melanoma specimens are acquired using fine needle aspiration biopsy [41]. However, currently, the two most commonly used treatments of uveal melanoma are radiation therapy and enucleation [42]. Enucleation remains the standard method of management of most large melanomas of the choroid and ciliary body. After enucleation, immunohistochemical assessment is relatively inexpensive and applicable to paraffin sections. High levels of HMGA1 expression may be linked to high proliferation rates of tumor cells by modulating chromatin structure and gene expression through DNA binding [20], [21]. There is increasing evidence suggesting that in some tumors, nuclear expression of HMGA1 was revealed a strong correlation with tumor grade and inversely associated with survival [43]–[45], HMGA1 protein can be used as a marker for metastatic progression [29]. Our previous study [28] found that high HMGA1 protein levels correlated with poor retinoblastoma prognosis. Here, we explored the correlation of HMGA1 levels with survival of UM patients. Adjusted for the largest basal tumor diameter, tumor thickness and epithelioid cell pattern, high level expression of HMGA1 was found to be associated independently with an increased risk of distant metastasis, and with shorter UM specific survival. Our data imply that HMGA1 protein is a reliable predictor for metastatic progression of UM. Furthermore, other tumor markers, such as Ki67 LI, mitoses counts and epithelioid cell pattern, which we found to be correlated with high HMGA1 expression levels in this study cohort, were also strongly correlated with metastatic mortality. This observation strengthened the role of HMGA1 protein as marker for UM metastatic prognosis.

Traditionally, prognostic markers for metastatic death from UM included the age of the patient and a largest basal diameter and an increasingly complex vascular pattern of the primary tumor [11], [14], [35], [46], [47]. A previous study [13] showed UM-specific mortality of 28.9% during a median follow-up period of 4.9 years. In our patient cohort, the mortality rate (27.8%; 24 death) during a median follow-up period of 55 months was similar despite the fact that the tumors were large (average basal diameter 14±3.4 mm) and thick (average 10.1±3.3 mm), the patients were on average younger (46±14.5 years). Evidently, the parameters used so far to predict disease outcome in this Chinese UM cohort may not be the most reliable. It is in this context that we believe that HMGA1 expression levels as determined in the present study may serve as a valuable prognostic marker.

Although much progress has been made in the diagnosis and early local treatment of UM, survival rates have not improved. Once metastases occur, prognosis is poor. In our study, as in previous ones [7], [8], the average life expectancy from diagnosis of metastasis to death was 5 months. Because HMGA1 may not only serve as a prognostic marker but may also be causally involved in metastatic progression, and because HMGA1 is not present in most adult normal tissues [20], [21], it may become an attractive target for cancer therapy [25], [48]. In fact, in pancreatic adenocarcinoma, blocking HMGA protein synthesis reduced tumor cell proliferation and metastatic potential, brought about a reduction in the Ki67 LI and caused an increase in the level of apoptosis [49]. Recently, Palmieri et al [50] reported that downregulation of HMGA-targeting miRNAs could contribute to increase HMGA protein levels in human pituitary adenomas and lead to pituitary tumorigenesis. In our previous study [28], we identified miRNAs that were dysregulated by *HMGA1* in retinoblastoma, further supporting the notion that HMGA1 is not only a marker for aggressive UM but also actively involved in gene regulation in this tumor. Based on our current findings, inhibiting HMGA1 protein expression may thus offer the possibility to prevent or delay metastases of UM without damaging normal tissues.

In conclusion, high levels of HMGA1 protein correlate with poor survival and might therefore serve as a prognostic predictor of UM progression. Additional studies will show whether HMGA1 protein might provide a potential therapeutic target for preventing or delaying metastases of UM.

## Supporting Information

Table S1 **Multivariate logistic regression analysis to assess relationship between expressions of epithelioid cells, mitoses count, Ki67 labeling index and HMGA1 status in uveal melanomas.** Multivariate logistic regression analysis assessed the relationship of the presence of epithelioid cells, mitoses count, and Ki67 labeling index with HMGA1 expression status in uveal melanomas, initially included age of diagnosis and sex. * Tumor showed HMGA1-high. 1, This cut-off is same as in Table 1 and 2; 2, The median of percentage of HMGA1 expression is “0”. This cut-off is HMGA1 stained *vs.* non-stained; 3, 75% percentage of HMGA1 expression is “20%”. This cut-off is >20% *vs.* ≤20%. CI, confidence interval; OR, odds ratio.
(DOC)
