# Supplementary material for: Overexpression of High Mobility Group A1 Protein in Human Uveal Melanomas: Implication for Prognosis
Source: PLoS One. 2013 Jul 23;8(7):e68724. doi: 10.1371/journal.pone.0068724 (PMC3720810; doi:10.1371/journal.pone.0068724)
Supplement: Table S1 — Multivariate logistic regression analysis to assess relationship between expressions of epithelioid cells, mitoses count, Ki67 labeling index and HMGA1 status in uveal melanomas. Multivariate logistic regression analysis assessed the relationship of the presence of epithelioid cells, mitoses count, and Ki67 labeling index with HMGA1 expression status in uveal melanomas, initially included age of diagnosis and sex. * Tumor showed HMGA1-high. 1, This cut-off is same as in Table 1 and 2; 2, The median of percentage of HMGA1 expression is “0”. This cut-off is HMGA1 stained vs. non-stained; 3, 75% percentage of HMGA1 expression is “20%”. This cut-off is >20% vs. ≤20%. CI, confidence interval; OR, odds ratio. (DOC) [file pone.0068724.s001.doc]

| Variable independently associated with | Multivariate OR (95% CI) | P value |
| --- | --- | --- |
| HMGA1* (high: 2+,3+,4+; n = 25)^1^ |  |  |
| Epithelioid cells + | 0.38 (0.05-2.70) | 0.33 |
| Mitoses count > 4 | 15.8 (2.87-86.6) | 0.0015 |
| Ki67 labeling index > 2 | 3.14 (0.90-10.9) | 0.07 |
| HMGA1* (high: 1+,2+,3+,4+; n = 29)^2^ |  |  |
| Epithelioid cells + | 2.62 (0.38-17.9) | 0.33 |
| Mitoses count > 4 | 6.01 (1.31-28.2) | 0.02 |
| Ki67 labeling index > 2 | 2.97 (0.84-10.54) | 0.09 |
| HMGA1* (high: > 20%; n = 21)^3^ |  |  |
| Epithelioid cells + | 0.64 (0.10-4.25) | 0.65 |
| Mitoses count > 4 | 12.4 (2.48-62.2) | 0.002 |
| Ki67 labeling index > 2 | 4.91 (1.35-17.9) | 0.016 |
